# Evaluating cross-resistance and synergy between Vip3Aa and Cry proteins from Bt in six strains of *Helicoverpa zea* derived via F_2_ screens

**DOI:** 10.1007/s44297-026-00073-8

**Published:** 2026-04-09

**Authors:** Yucheng Wang, David L. Kerns, Graham P. Head, Dawson Kerns, Bruce E. Tabashnik, Fei Yang

**Affiliations:** 1 Department of Entomology, University of Minnesota, St. Paul, MN USA; 2 Department of Entomology, Texas A&M University, College Station, TX USA; 3 Bayer Crop Science, St. Louis, MO USA; 4 Department of Entomology, Louisiana State University, Macon Ridge Research Station, Winnsboro, LA USA; 5 Department of Entomology, University of Arizona, Tucson, AZ USA

**Keywords:** Corn earworm, Resistance management, Transgenic crops, *Bacillus thuringiensis*, Cross-resistance, Synergy

## Abstract

Transgenic crops producing insecticidal *Bacillus thuringiensis* (Bt) proteins have revolutionized the control of some pests. However, the evolution of resistance by pests has reduced the efficacy of Bt crops. Pest resistance to Bt crystalline (Cry) proteins has spurred a shift to crops that produce the Bt vegetative insecticidal protein Vip3Aa together with Cry proteins. With widespread field-evolved practical resistance of the major pest *Helicoverpa zea* (corn earworm or bollworm) to Cry proteins in the United States, Vip3Aa is the only Bt protein in transgenic corn and cotton that is highly effective against many populations. We generated five strains of *H. zea* with > 100-fold resistance to Vip3Aa in a susceptible genetic background using F_2_ screens of field populations from Louisiana, Mississippi, and Texas followed by crosses to a susceptible strain and additional laboratory selection with Vip3Aa. The results from diet bioassays show that in these Vip3Aa-resistant strains, cross-resistance to Cry1Ab, Cry1Ac, Cry1A.105, Cry1Fa, and Cry2Ab was not consistent and ranged 1100-fold: from 16-fold negative cross-resistance to 65-fold positive cross-resistance. By crossing a Vip3Aa-resistant strain with a Cry-resistant strain followed by selection with both types of Bt protein, we generated a strain highly resistant to Cry1Ac, Cry2Ab, and Vip3Aa. Cry1Ac and Cry2Ab interacted synergistically with Vip3Aa against this triple-resistant strain, such that combinations of the Cry proteins and Vip3Aa caused higher mortality than expected from their individual toxicity. The observed synergy may increase the durability of Bt crops producing combinations of these proteins against *H. zea*.

**Supplementary Information:**

The online version contains supplementary material available at 10.1007/s44297-026-00073-8.

## Introduction

Transgenic crops producing *Bacillus thuringiensis* (Bt) proteins have greatly advanced pest management [1–4], but their widespread adoption has selected for the evolution of resistance by pests. Practical resistance, defined as field-evolved resistance that diminishes efficacy and has practical consequences for pest control [5], has been documented in at least 35 cases involving 11 species of Lepidoptera and Coleoptera in eight countries [6–12]. Nearly all documented cases of practical resistance to Bt crops involve crystalline (Cry) Bt proteins, which are produced by crops that were first commercialized in 1996 and have been planted on a cumulative total of 1.8 billion hectares worldwide [11].

Practical resistance to Cry proteins has spurred a shift to multi-toxin Bt crops that produce Cry proteins together with the Bt vegetative insecticidal protein Vip3Aa, which is generally effective against some major lepidopteran pests that are resistant to Cry proteins [13–15]. For *Helicoverpa zea* (corn earworm or bollworm), one of the most economically important crop pests in the United States [16–19], practical resistance to Vip3Aa has been reported in Brazil [6], and several lines of evidence provide early warning of resistance to Vip3Aa in the United States [20–23]. Because Bt crops that produce Vip3Aa also produce one or more Cry proteins targeting lepidopterans, a better understanding of cross-resistance and synergy between Vip3Aa and Cry proteins may be useful for sustaining the efficacy of such multi-toxin Bt crops.

Binding of Bt proteins to midgut receptors is required for toxicity; disruption of this binding is the most common and most potent mechanism of resistance [24]. Selection with one Bt protein that causes resistance to that protein by disrupting a midgut receptor essential for toxicity of that protein and other closely related Bt proteins can cause strong cross-resistance to the other Bt proteins [25–27]. Because Vip3Aa and Cry proteins do not bind to the same midgut receptors, strong cross-resistance between these toxins is not expected [28].

Despite the ideas noted above, cross-resistance between distantly related Bt proteins that do not share receptors can occur when reduced binding is not the primary mechanism of resistance [26]. For example, in a landmark study that has been cited nearly 500 times, laboratory selection of *Chloridea virescens* with Cry1Ac yielded 50-fold resistance to Cry1Ac that was not associated with reduced binding of Cry1Ac, as well as 53-fold cross-resistance to Cry2Ab, which does not share receptors with Cry1Ac [29]. Notably, reduced binding of Vip3Aa is not the primary mechanism of resistance to Vip3Aa in several cases [30]. Additionally, a review of 29 cases that rigorously compared related strains in seven species of lepidopteran pests revealed overall statistically significant, weak cross-resistance between Cry1 and Vip3 proteins (n = 21 cases) but no consistent cross-resistance between Cry2A and Vip3 proteins (n = 8 cases) [28].

In addition to cross-resistance, interactions among the different Bt proteins in each multi-toxin plant can influence the efficacy of Bt crops. Of 157 diet bioassay evaluations of combinations of Cry and Vip proteins against lepidopterans reviewed in 2020, only 19% of the interactions were synergistic [31]. However, synergism between Cry and Vip proteins was much more common in several studies published after 2020 [32–35], including 89% of 45 cases for *Spodoptera frugiperda* [35] and 100% of 30 cases for *Conogethes punctiferalis* [34]. As far as we know, previous evaluations of synergy between Cry and Vip proteins used insect strains that were susceptible to Cry and Vip proteins, with one exception. An exceptional study found independent action of Cry1Ac and the chimeric protein Vip3AcAa against two strains of *Helicoverpa armigera* that were resistant to Cry1Ac and susceptible to Vip3AcAa [36]. While the previous data are primarily useful for understanding the efficacy of multi-toxin combinations against susceptible populations, data on interactions between Cry and Vip proteins against insects resistant to Cry and Vip proteins are critical for understanding, monitoring, and managing resistance.

Here, we focus on cross-resistance and synergy between Vip3Aa and Cry proteins in *H. zea*. In the United States, widespread practical resistance of *H. zea* to Cry proteins in Bt corn and cotton has been documented, while Vip3Aa has largely retained its field efficacy against this polyphagous lepidopteran [14, 20, 37–42]. These field efficacy results are consistent with the expectation of no strong cross-resistance to Vip3Aa caused by resistance to Cry proteins based on their lack of shared binding sites as well as data from previous studies of three laboratory-selected strains of *H. zea* [43–45]. However, cross-resistance is not always symmetrical between Bt proteins [46, 47], and we are aware of only one previous study that tested *H. zea* for cross-resistance to Cry proteins caused by selection with Vip3Aa [48]. Whereas previous results with Bt-susceptible *H. zea* suggest that synergy occurred between Cry1Ab and Vip3Aa on plants of Bt corn but not Bt cotton [49, 50], we did not find previous publications evaluating synergy between Cry and Vip proteins against Bt-resistant *H. zea*.

Here, we evaluated *H. zea* cross-resistance to five Cry proteins in five strains that were initially isolated via F_2_ screens of field populations for resistance to Vip3Aa and further selected with Vip3Aa in the laboratory. We also evaluated synergy among Cry1Ac, Cry2Ab, and Vip3Aa in a strain that was resistant to all three of these Bt proteins. The results show 1100-fold variation among Vip3Aa strains in their cross-resistance to Cry proteins, ranging from 17-fold negative cross-resistance to 65-fold positive cross-resistance. We also discovered synergy between Cry proteins and Vip3Aa, which could help to delay the evolution of *H. zea* resistance to Vip3Aa.

## Results

### Temporal variation in a susceptible strain’s responses to Vip3Aa and Cry proteins

To test for temporal variation in responses to Bt proteins in the susceptible strain BZ-SS, we tested it against Vip3Aa, Cry1Ac, and Cry2Ab in October 2020, January 2021, and July 2021. The maximum variation among LC_50_ values of the same Bt protein measured at these three different times was 3.0-fold for Vip3Aa, 4.1-fold for Cry1Ac, and 6.4-fold for Cry2Ab (Tables 1 and 2). Based on the mean of the log-transformed LC_50_ values after back-transformation, the mean LC_50_ values (in μg Bt protein per cm^2^ diet) for BZ-SS were 0.18 for Vip3Aa, 0.06 for Cry1Ac, and 0.24 for Cry2Ab.

**Table 1:** Responses of *H. zea* to Vip3Aa in diet bioassays

Strain	Date	Slope ± SE ^a^	LC_50_ (95% FL) ^b^	RR ^c^
BZ-SS	October 2020	3.2 ± 0.4	0.11 (0.09, 0.13)	0.6
BZ-SS	January 2021	2.8 ± 0.3	0.17 (0.14, 0.21)	0.9
BZ-SS	July 2021	1.8 ± 0.4	0.33 (0.16, 0.78)	1.8
LT70-Vip	December 2020	NA ^d^	> 100	> 500
AC4-Vip	October 2021	NA	> 100	> 500
M1-Vip	June 2021	NA	> 100	> 500
R2-Vip	September 2021	NA	> 100	> 500
R15-Vip	September 2021	NA	> 100	> 500

^a^Slope of the concentration-mortality line and standard error (SE) of the slope

^b^Concentration killing 50% of larvae (LC_50_) and its 95% fiducial limits (FL) in μg Vip3Aa per cm^2^ diet; n = 448 to 452 larvae tested for each LC_50_

^c^Resistance ratio (RR) = LC_50_ for a strain divided by the mean LC_50_ for BZ-SS (0.18) based on the back-transformed mean of log (LC_50_)

^d^Not available because the mortality at the highest concentration tested (100 μg Vip3Aa per cm^2^ diet) was 0 to 15%

**Table 2:** Responses of *H. zea* to five crystalline (Cry) proteins in diet bioassays

Bt protein	Strain	Date	Slope ± SE ^a^	LC_50_ (95% FL) ^b^	CRR ^c^
Cry1Ab	BZ-SS	October 2021	1.5 ± 0.2	0.035 (0.022, 0.051)	1.0
	LT70-Vip	September 2021	1.1 ± 0.2	0.006 (0.002, 0.010)	0.2*
	AC4-Vip	October 2021	0.8 ± 0.1	0.17 (0.12, 0.25)	4.9*
	M1-Vip	June 2021	1.3 ± 0.2	0.017 (0.011, 0.024)	0.5
	R15-Vip	November, 2021	1.6 ± 0.3	0.006 (0.002, 0.009)	0.2*
Cry1Ac	BZ-SS	October 2020	1.6 ± 0.2	0.027 (0.020, 0.035)	0.4
	BZ-SS	January 2021	1.4 ± 0.1	0.11 (0.08, 0.14)	1.7
	BZ-SS	July 2021	1.2 ± 0.1	0.09 (0.07, 0.12)	1.4
	LT70-Vip	December 2020	1.1 ± 0.1	0.17 (0.12, 0.23)	2.6
	AC4-Vip	October 2021	1.0 ± 0.1	1.6 (1.1, 2.2)	24*
	M1-Vip	June 2021	1.4 ± 0.1	0.60 (0.46, 0.78)	9.3*
	R2-Vip	December 2021	0.9 ± 0.1	1.0 (0.73, 1.4)	16*
	R15-Vip	November 2021	2.3 ± 0.3	0.05 (0.04, 0.07)	0.8
Cry1A.105	BZ-SS	October 2020	2.6 ± 0.3	0.025 (0.020, 0.030)	1.0
	LT70-Vip	December, 2020	1.7 ± 0.3	0.006 (0.002, 0.009)	0.2*
	AC4-Vip	October, 2021	0.8 ± 0.1	1.6 (0.95, 3.2)	65*
	M1-Vip	June, 2021	1.8 ± 0.2	0.026 (0.018, 0.037)	1.0
	R15-Vip	November, 2021	1.5 ± 0.3	0.006 (0.003, 0.009)	0.2*
Cry1Fa	BZ-SS	October 2021	0.9 ± 0.1	8.36 (5.74, 13.23)	1.0
	LT70-Vip	September 2021	1.5 ± 0.1	0.53 (0.40, 0.68)	0.06*
	AC4-Vip	October 2021	0.9 ± 0.1	33.2 (19.0, 76.0)	3.9*
	M1-Vip	June 2021	0.9 ± 0.1	0.57 (0.31, 0.93)	0.07*
	R2-Vip	December 2021	1.2 ± 0.2	19.8 (11.7, 44.2)	2.4
	R15-Vip	November 2021	1.8 ± 0.2	0.85 (0.67, 1.1)	0.1*
Cry2Ab	BZ-SS	October 2020	1.8 ± 0.2	0.18 (0.14, 0.23)	0.8
	BZ-SS	January 2021	1.5 ± 0.2	0.70 (0.51, 0.97)	2.9
	BZ-SS	July 2021	1.3 ± 0.1	0.11 (0.08, 0.16)	0.5
	LT70-Vip	December 2020	1.4 ± 0.1	0.34 (0.26, 0.44)	1.4
	AC4-Vip	October 2021	1.0 ± 0.1	6.0 (3.5, 12.0)	25*
	M1-Vip	June 2021	1.0 ± 0.1	0.098 (0.070, 0.13)	0.4
	R2-Vip	December 2021	1.6 ± 0.3	0.35 (0.21, 0.59)	1.5
	R15-Vip	November 2021	1.7 ± 0.2	0.22 (0.16, 0.31)	0.9

^a^Slope of the concentration-mortality line and standard error (SE) of the slope

^b^Concentration killing 50% of larvae (LC_50_) and its 95% fiducial limits (FL) in μg *Bacillus thuringiensis* (Bt) protein per cm^2^ diet; n = 448 to 576 (mean = 496) larvae tested for each LC_50_

^c^Cross-resistance ratio (CRR) = LC_50_ for a strain divided by the mean LC_50_ for BZ-SS tested against the same Bt protein; mean for BZ-SS based on back-transformed mean of log (LC_50_): 0.06 for Cry1Ac, 0.24 for Cry2Ab, and 0.18 for Vip3Aa in μg Bt protein per cm^2^ diet

^*^LC_50_ significantly different between a Vip-RR strain and BZ-SS based on non-overlap of the 95% FL

### Crosses to BZ-SS and laboratory selection with Vip3Aa yield resistance to Vip3Aa in the BZ-SS genetic background

After five to six rounds of backcrossing to BZ-SS and selection with Vip3Aa, the mortality of the five Vip3-resistant strains (LT70-Vip, AC4-Vip, M1-Vip, R2-Vip and R15-Vip) caused by the highest concentration of Vip3Aa tested (100 μg Vip3Aa per cm^2^ diet) was only 0 to 15%. Thus, we infer that the LC_50_ values were > 100 μg Vip3Aa per cm^2^ diet. Furthermore, the Vip3Aa resistance ratio (RR, the LC_50_ value for each strain divided by the mean LC_50_ value for BZ-SS for Vip3Aa) was > 500 (> 100 divided by 0.18, the mean LC_50_ value of Vip3Aa for BZ-SS, Table 1).

### Variable cross-resistance to Cry proteins caused by selection with Vip3Aa

**Fig. 1:**
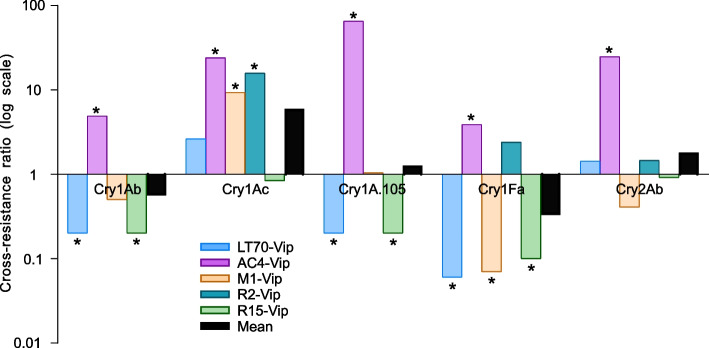
Cross-resistance ratios for five crystalline proteins in five Vip3Aa-resistant strains of *H. zea*. * indicates an LC_50_ that is significantly different between a Vip3Aa-resistant strain and the susceptible strain BZ-SS based on non-overlap of the 95% FL (see Methods)

Overall, the responses of five strains of *H. zea* selected for resistance to Vip3Aa do not show consistent positive or negative cross-resistance to the five Cry proteins tested (Cry1Ab, Cry1Ac, Cry1A.105, Cry1Fa, and Cry2Ab; Fig. 1 and Table 2). Seven of the 23 LC_50_ values of Cry proteins were significantly higher than those of BZ-SS, seven were significantly lower, and nine did not differ significantly from those of BZ-SS (Fig. 1 and Table 2). The 7:7 ratio of cases with significant positive and negative cross-resistance matches the 1:1 ratio expected if no consistent cross-resistance occurs.

We calculated the cross-resistance ratio (CRR) as the LC_50_ value for each strain divided by the mean LC_50_ of BZ-SS for the same Cry protein. The 23 LC_50_ values yield a mean CRR for Cry proteins of 1.2 (95% CI = 0.5 to 3.0, Table 3). This mean CRR does not differ significantly from the CRR of 1.0 expected if selection with Vip3Aa did not cause cross-resistance to Cry proteins (one-sample t-test of log-transformed data, *t* = 0.52, *df* = 22, *P* = 0.61).

**Table 3:** Mean cross-resistance to five crystalline (Cry) proteins across *H. zea* strains resistant to Vip3Aa

Cry protein	n ^a^	Mean CRR ^b^	95% CI ^c^	*P* ^d^
Cry1Ab	4	0.6	0.05 to 6.2	0.50
Cry1Ac	5	6.0	1.1 to 33	0.04
Cry1A.105	4	1.3	0.02 to 98	0.87
Cry1Fa	4	0.3	0.03 to 4.2	0.29
Cry2Ab	5	1.8	0.3 to 12	0.44
All Cry1	18	1.1	0.4 to 3.2	0.82
All Cry	23	1.2	0.5 to 3.0	0.61

^a^Number of evaluated cross-resistance ratios (CRRs)

^b^Based on the mean of log-transformed CRRs (Table 2), then back-transformed

^c^95% confidence interval (CI) of mean CRR

^d^Probability from one-sample t-test of the observed results if the null hypothesis of CRR = 1 was true

Despite the lack of significant overall cross-resistance to Cry proteins in the five Vip3Aa-resistant strains, the 23 CRRs for Cry proteins varied 1100-fold: from 0.06 for LT70-Vip for Cry1Fa to 65 for AC4-Vip for Cry1A.105 (Table 2). Of the 23 CRRs for Cry proteins in Vip3Aa-resistant strains, four CRRs in two strains (AC4-Vip and R2-Vip) were > 10, while three CRRs in the three other strains were 0.1 or less, each reflecting significant negative cross-resistance of at least tenfold to Cry1Fa (Table 2).

Using one-sample t-tests to evaluate cross-resistance caused by selection with Vip3Aa separately for each Cry protein revealed significant cross-resistance to Cry1Ac (mean CRR = 6.0, 95% CI = 1.1 to 33, *t* = 2.9, *df* = 4, *P* = 0.04) and no significant positive or negative cross-resistance for each of the four other Cry proteins tested (mean CRR = 0.3 to 1.8, *P* > 0.28 for each, Table 3). Additionally, analyzing the four Cry1 proteins together revealed no significant overall cross-resistance caused by selection with Vip3Aa (Table 3).

Analyzing each of the five Vip3Aa-resistant strains separately for cross-resistance to Cry proteins indicated significant positive cross-resistance in AC4-Vip (mean CRR = 15, 95% CI = 3.4 to 66, *t* = 5.1, *df* = 4, *P* = 0.007) and no significant positive or negative cross-resistance for each of the four other strains (*P* = 0.06 to 0.64, Table 4). The mean CRR for Cry proteins was 0.4 for LT70-Vip and 0.7 for M1-Vip, which is consistent with the reduced binding of Vip3Aa to midgut brush border membrane vesicles (BBMVs) in LT70-Vip [51] and the shared resistance locus between these two strains [52]. However, the mean CRR was 13 times higher for R2-Vip (3.8) than R15-Vip (0.3; t-test, *t* = 3.2, df = 6, *P* = 0.02), which is surprising because these two strains also share a resistance locus [51].

**Table 4:** Mean cross-resistance across crystalline (Cry) proteins for each of five strains of *H. zea* resistant to Vip3Aa

Strain	n ^a^	Mean CRR ^b^	95% CI ^c^	*P* ^d^
TX-LT70-Vip	5	0.4	0.1 to 2.7	0.25
LA-AC4-Vip	5	15	3.4 to 66	0.01
LA-M1-Vip	5	0.7	0.1 to 6.0	0.64
MS-R2-Vip	3	3.8	0.2 to 87	0.21
MS-R15-Vip	5	0.3	0.1 to 1.1	0.06

^a^Number of Cry proteins tested per strain

^b^Mean cross-resistance ratio (CRR) calculated from the mean of log-transformed resistance ratios (RRs) (Table 2) and then back-transformed

^c^95% confidence interval of mean CRR

^d^Probability from one-sample t-test of the observed results if the null hypothesis of CRR = 1 was true

Whereas the results summarized above are based on CRRs calculated using as the divisor the mean LC_50_ of BZ-SS for the same Cry protein, the overall results are nearly identical using as the divisor the single LC_50_ of BZ-SS obtained closest to the date each Vip3Aa-resistant was tested with the same Cry protein. With this alternative method, the 23 LC_50_ values yield a mean CRR for Cry proteins of 1.2 (95% CI = 0.5 to 3.0; back-transformed values from analysis of log-transformed data), which does not differ significantly from the CRR of 1.0 expected if selection with Vip3Aa did not cause cross-resistance to Cry proteins (one-sample t-test of log-transformed data, *t* = 0.54, *df* = 22, *P* = 0.59). Using this method produces the identical 1100-fold range noted above for the 23 RRs (0.06 to 65).

### Synergy between Vip3Aa and Cry1Ac or Cry2Ab against a strain resistant to all three of these Bt proteins

The triple-resistant (TRE-RR) strain had RRs of 265 for Cry1Ac, 495 for Cry2Ab, and > 96 for Vip3Aa (Table 5). For TRE-RR, synergy between Vip3Aa and Cry1Ac occurred at both concentrations evaluated (3.16 and 5 μg Bt protein per cm^2^ diet), as indicated by significantly lower observed survival for these proteins combined than expected based on survival with each tested separately (Table 6). For Cry2Ab, synergy with Vip3Aa occurred at a concentration of 5 but not 3.16 μg Bt protein per cm^2^ diet (Table 6). With all three Bt proteins combined, synergy occurred at both concentrations evaluated based on significantly lower observed survival than expected based on survival with each Bt protein tested separately (Table 6).

**Table 5:** Responses of a triple-resistant (TRE-RR) and susceptible (BZ-SS) strain of *H. zea* to Cry1Ac, Cry2Ab, and Vip3Aa in diet bioassays

Bt protein	Strain	Slope ± SE ^a^	LC_50_ (95% FL) ^b^	RR ^c^
Cry1Ac	BZ-SS	1.2 ± 0.1	0.09 (0.07, 0.12)	1.0
	TRE-RR	0.8 ± 0.1	23.8 (10.7, 109)	265
Cry2Ab	BZ-SS	1.3 ± 0.1	0.11 (0.08, 0.16)	1.0
	TRE-RR	0.7 ± 0.2	54.5 (20.9, 420)	495
Vip3Aa	BZ-SS	1.8 ± 0.4	0.33 (0.16, 0.78)	1.0
	TRE-RR	NA ^d^	> 31.6	> 96

^a^Slope of the concentration-mortality line and its standard error (SE)

^b^Concentration killing 50% of larvae (LC_50_) and its 95% fiducial limits (FL) in μg *Bacillus thuringiensis* (Bt) protein per cm^2^ diet; n = 512 larvae tested for each strain against each Bt protein, except n = 384 for Cry1Ac and 448 for Cry2Ab for TRE-RR

^c^Resistance ratio = LC_50_ divided by the LC_50_ of BZ-SS for the same Bt protein. The RRs for TRE-RR, but not other details, were reported in Calvin et al. 2024

^d^Not available because the mortality at the highest concentration tested (31.6 μg Vip3Aa per cm^2^ diet) was 3%

**Table 6:** Synergy between crystalline (Cry) proteins and Vip3Aa against the triple-resistant strain TRE-RR of *H. zea*

Bt protein(s)	Concn. ^a^	Observed survivors (O)^ b^	Expected surviors (E) ^c^	(O-E)/E	*χ*^2^	*P* ^d^
Cry1Ac	3.16	51	NA	NA	NA	NA
Cry1Ac	5.00	58	NA	NA	NA	NA
Cry2Ab	3.16	36	NA	NA	NA	NA
Cry2Ab	5.00	45	NA	NA	NA	NA
Vip3Aa	3.16	63	NA	NA	NA	NA
Vip3Aa	5.00	63	NA	NA	NA	NA
Cry1Ac + Vip3Aa	3.16	41	50.2	−0.18	7.0	0.008
Cry1Ac + Vip3Aa	5.00	42	57.1	−0.26	34.6	< 0.0001
Cry2Ab + Vip3Aa	3.16	35	35.4	−0.01	0.0	1
Cry2Ab + Vip3Aa	5.00	19	44.3	−0.57	45.1	< 0.0001
Cry1Ac + Cry2Ab + Vip3Aa	3.16	18	28.2	−0.36	6.0	0.01
Cry1Ac + Cry2Ab + Vip3Aa	5.00	9	40.1	−0.78	62.7	< 0.0001

^a^Concentration in μg *Bacillus thuringiensis* (Bt) protein per cm^2^ diet

^b^Total larvae tested = 64 per row

^c^Expected survival based on independent action

^d^Probability from the chi-squared test that the observed outcome occurred if the null hypothesis of no difference between the number of observed and expected survivors was true

For Bt proteins tested singly or in combination, survival was not consistently lower at 5 μg of Bt protein per cm^2^ diet than at 3.16 μg of Bt protein per cm^2^ diet (Table 6). For each Bt protein, the same batch was used on both dates, and synergy bioassays were conducted. However, because each concentration was tested on a different date, the variation in survival between concentrations might reflect differences between dates in the larvae, diet, or both used in the bioassays. Analysis of synergy was not affected by variation between dates because the efficacy of Bt proteins singly versus in combinations was compared on the same date for each of the two concentrations.

## Discussion

We found that selection of *H. zea* with vegetative insecticidal protein Vip3Aa did not consistently cause positive or negative cross-resistance to crystalline (Cry) proteins. The mean cross-resistance ratio (CRR) based on 23 evaluations of cross-resistance to Cry proteins in five Vip3Aa-resistant strains of *H. zea* was 1.2, which does not support a previous suggestion that negative cross-resistance generally occurs between Vip3Aa and Cry proteins [30, 48]. The five Vip3Aa-resistant strains studied here were started from F_2_ screens of field populations that were probably resistant to Cry proteins. Thus, we cannot exclude the presence of alleles conferring resistance to Cry proteins that were selected in the field, which would tend to increase CRRs for Cry proteins and bias this evaluation. However, each Vip3Aa-resistant strain was crossed at least five times with the BZ-SS strain and then reselected with Vip3Aa, which minimized this potential bias. Five crosses to BZ-SS are expected to replace the genetic background of the field populations with the BZ-SS genetic background for 97% of loci not directly causing resistance to Vip3Aa or tightly linked with genes causing resistance to Vip3Aa.

Of the five Vip3a-resistant strains analyzed here, the physiological mechanism of resistance has been determined only for LT70-Vip, which is reduced binding of Vip3Aa to midgut brush border membrane vesicles (BBMVs) [51]. As expected with this mechanism, LT70-Vip showed no significant cross-resistance to Cry proteins. Interstrain complementation results implying that the alleles conferring resistance to Vip3Aa occur at the same locus in M1-Vip and LT70-Vip [52] suggest that reduced binding of Vip3Aa may also be a key resistance mechanism in M1-Vip. Although the overall CRR for Cry proteins was 0.7 for M1-Vip, the LC_50_ of Cry1Ac was 9.3 times greater in M1-Vip than BZ-SS. The statistically significant resistance to Cry1Ac in M1-Vip relative to BZ-SS could reflect cross-resistance or, despite the repeated crosses to BZ-SS, the presence of alleles conferring resistance to Cry proteins that were selected in the field. In general, mechanisms other than reduced binding of Bt proteins to larval midgut membranes, such as altering the processing of Bt proteins, can confer broad cross-resistance [24–29]. Determining the mechanism of resistance to Vip3Aa in the other four Vip3Aa-resistant strains examined here could help to address this issue, advance understanding of the apparent positive overall cross-resistance in AC4-Vip (mean CRR = 15, range 3.9 to 65), and clarify why cross-resistance differed between R2-Vip and R15-Vip despite their shared resistance locus, as indicated by complementation results [52].

Most previous studies evaluating cross-resistance between Cry and Vip3Aa proteins, including all 29 cases analyzed by Tabashnik and Carrière [28], avoided the potential contribution of multiple selection that arose in this study from comparing the susceptible strain BZ-SS to Vip3Aa-resistant strains derived from field populations that were probably resistant to Cry proteins. The more direct approach in most other studies tested for cross-resistance to a Vip3 protein by comparing susceptibility to that Vip3 protein between a strain selected in the laboratory with a Cry protein and its parent unselected strain (or conversely, tested for cross-resistance to a Cry protein by comparing susceptibility to that Cry protein between a strain selected in the laboratory with a Vip3 protein and its parent unselected strain). Thus, multiple selection did not contribute to the previous finding of weak, statistically significant cross-resistance between Cry1 and Vip3 proteins [28].

In addition, we analyzed the 11 cases summarized in Table 1 of Roy et al. [30], where the direct lab selection approach (rather than F_2_ screens of field populations) was applied to three other species of noctuid moths: *Chloridea virescens, Mythimna separata,* and *Spodoptera frugiperda*. The results reveal that standard lab selection with Vip3Aa caused overall weak, statistically significant cross-resistance to Cry proteins (one-sample t-test of log-transformed CRRs, mean CRR = 1.7, *t* = 2.7, df = 10, *P* = 0.02). The conclusion is similar based on the 9 of 11 aforementioned cases published after 2020 that were not included in the analysis by Tabashnik and Carrière [28] (one-sample t-test of log-transformed CRRs, mean CRR = 1.5, *t* = 2.6, df = 8, *P* = 0.03).

Nonetheless, for the Vip3Aa-resistant strain of *S. frugiperda* derived from an F_2_ screen of a field population from Rapides Parish, Louisiana in 2016, the CRRs for three Cry proteins decreased after an additional cross to a susceptible strain, additional laboratory selection with Vip3Aa, and no exposure to Cry proteins in the laboratory [53]. Thus, the observed decrease in CRRs for Cry proteins could reflect fitness costs of Cry resistance [54], negative cross-resistance, and/or loss of field-derived Cry resistance alleles caused by the additional cross to the susceptible strain.

The 1100-fold variation in the range of positive and negative cross-resistance to Cry proteins for Vip3Aa-resistant strains of *H. zea* in this study (0.06 to 65) exceeds the ranges reported previously for cross-resistance between Vip3 and Cry proteins. Unlike previous studies, 4 of the 23 CRRs for cross-resistance to Cry proteins in Vip3Aa-resistant strains of *H. zea* indicate > tenfold positive cross-resistance, while 3 show at least tenfold negative cross-resistance. In contrast, the 20 CRRs evaluating cross-resistance to Cry proteins in Vip3Aa-resistant strains of five other species of noctuid moths reviewed by Roy et al. [30] were only 0.3 to 6.7, which is 22-fold. Moreover, in the 29 CRRs reviewed by Tabashnik and Carrière [28] to evaluate cross-resistance to Vip3 caused by selection with Cry proteins (n = 21) and vice versa (n = 8) in seven lepidopteran pests, the range was only 15-fold (0.3 to 4.6). Thus, the range in CRRs testing for cross-resistance to Cry proteins in the five Vip3Aa-resistant strains of *H. zea* is 49 times greater than for the five other noctuids reviewed by Roy et al. [30] and 70 times greater than for the seven lepidopterans reviewed by Tabashnik and Carrière [28].

One factor that might have contributed to the large range in the RRs for Cry proteins in the Vip3Aa-resistant strains of *H. zea* is the origin of each of these strains from F_2_ screens of field populations. However, this hypothesis is not supported by the results for five other moth species in Table 1 of Roy et al. [30]. For that data, the range in CRRs indicating cross-resistance to Cry proteins in Vip3Aa-resistant strains was not greater for nine strains generated via F_2_ crosses (10.7-fold, 0.3 to 3.2) than for 11 strains generated via standard lab selection (11.2-fold, 0.6 to 6.7). Another factor that could have contributed to the wide range in CRRs for Cry proteins is variation in the LC_50_ value of each Cry protein for BZ-SS, which is the divisor for RRs. However, for the two Cry proteins that were each tested on three dates against BZ-SS (Cry1Ac and Cry1Fa), the maximum range in LC_50_ of a particular Cry protein for BZ-SS was only 6.4-fold.

We found synergy in diet bioassays here between Vip3Aa and either Cry1Ac or Cry2Ab in the triple-resistant (TRE-RR) strain of *H. zea*, which was resistant to all three of these Bt proteins. The synergy seen here with TRE-RR is consistent with the synergy between Vip3Aa and Cry1Ab based on our analysis of previously reported data on larvae per ear from susceptible field populations of *H. zea* infesting Bt corn in 2007 and 2009 [49]. However, it remains to be determined whether similar synergy occurs for field-selected resistant *H. zea* on Bt corn or cotton in the field. If such synergy does occur, it would reduce the survival of resistant insects on Bt plants, thereby reducing the intensity of selection for resistance and potentially slowing the evolution of resistance.

If synergy occurs in the field against resistant insects, it might also have important implications for resistance monitoring. Currently, resistance is monitored with sentinel plots of corn producing Cry1Ab + Vip3Aa [23], which are used in part because plants producing Vip3Aa alone are not readily available. From such sentinel plots, the phenotypic frequency of resistance (PFR) in *H. zea* was calculated as larval abundance in ears of Cry1Ab + Vip3Aa corn divided by larval abundance in related non-Bt corn ears [23]. If synergy between Cry1Ab (which is similar to Cry1Ac) and Vip3Aa kills some larvae that are resistant to Cry1Ab alone and Vip3Aa alone, then the PFR calculated from this approach could underestimate the PFR for Vip3Aa. To clarify this issue, future work could compare the Vip3Aa resistance allele frequency and PFR estimated from F_2_ screens versus sentinel plots.

## Materials and Methods

### Insect strains

We used diet bioassays (see below) to test a susceptible strain and six strains of *H. zea* that were selected for resistance to *Bacillus thuringiensis* (Bt) in the laboratory (five selected with Vip3Aa and one selected with Cry1Ac, Cry2Ab, and Vip3Aa). The susceptible strain of *H. zea*, BZ-SS, was obtained from Benzon Research Inc. (Carlisle, PA, U.S.) in 2017 and reared without exposure to Bt proteins or other insecticides [20, 43, 55, 56]. Five Vip3Aa-resistant strains were started via F_2_ screens: CBW-TX-LT70-VIP-Vip (LT70-Vip) originated from Snook, Texas in 2019 [51, 52, 57]; CBW-LA-AC4-VIP-RR (AC4-Vip) from Winnsboro, Louisiana (LA) in 2020; CBW-LA-M1-VIP-RR (M1-Vip) from Alexandria, LA in 2019; CBW-MS-R2-VIP-RR (R2-Vip) and CBW-MS-R15-VIP-RR (R15-Vip) from Stoneville, MS in 2020 [52]. A Cry-resistant strain, CBW-TX-G13-Cry-RR (G13-Cry), was established via an F_2_ screen from insects sampled from Genuity VT Double Pro corn fields in Snook, TX in 2018 and selected in the lab with Cry1Ac and Cry2Ab [43, 55].

Before conducting bioassays to assess the susceptibility of the five Vip3Aa-resistant strains, each of these strains was backcrossed to BZ-SS at least five times and selected with Vip3Aa protein at least six times (Table S1-S5). The Cry-resistant strain (G13-Cry) was backcrossed to BZ-SS approximately seven times and selected with Cry1Ac and Cry2Ab more than 10 times. The backcrosses to BZ-SS followed by selection are expected to make the genetic background of the resistant strains similar to BZ-SS while maintaining the alleles conferring resistance to the Vip3Aa or Cry proteins used for selection. For example, after five backcrosses to BZ-SS with a Vip3Aa-resistant strain, the mean similarity with alleles from BZ-SS is expected to be 97% (1–0.5^5^) for genes that do not confer resistance to Vip3Aa and are not linked with genes conferring resistance to Vip3Aa. The five Vip3Aa-resistant strains had high levels of autosomal, functionally recessive resistance to Vip3Aa [55, 57, 58].

The results from interstrain complementation tests imply that alleles conferring resistance to Vip3Aa occur at three genetic loci in these five strains: one locus shared by LT70-Vip and M1-Vip, a second locus shared by R2-Vip and R15-Vip, and a third locus found only in AC4-Vip [52]. Based on these results, we hypothesized that cross-resistance to Cry proteins would be similar between LT70-Vip and M1-Vip as well as between R2-Vip and R15-Vip. Additionally, relative to the susceptible strain of *H. zea* from Benzon, the binding of Vip3Aa to midgut brush border membrane vesicles (BBMVs) was reduced in LT70-Vip (aka CEW-Vip-RR) [51]. Thus, we hypothesized that little or no cross-resistance to Cry proteins would occur in LT70 or in M1-Vip, which shares a Vip3Aa resistance locus with LT70-Vip.

To create the triple-resistant (TRE-RR) strain resistant to Cry1Ac, Cry2Ab, and Vip3Aa proteins, we conducted reciprocal crosses between the Vip3Aa-resistant strain LT70-Vip and the Cry-resistant strain G13-Cry. Adults were paired in 3.8-L paper containers (Huhtamaki Foodservice, De Soto, Kansas) containing ~ 100 g of vermiculite (Sun Gro, Pine Bluff, AR) and maintained in environmental chambers at 26 ± 1 °C, ∼60% relative humidity (RH), and a photoperiod of 16:8 h (L:D). F_1_ larvae obtained from the crosses were reared individually on Ward’s Stonefly Heliothis diet (Rochester, NY) in 30-ml plastic cups (Fill-Rite, Newark, NJ) until pupation. Pupae were transferred into paper containers for adult emergence, mating, and reproduction. Approximately 4,000 F_2_ neonates produced by F_1_ moths were selected for resistance to Cry1Ac + Cry2Ab + Vip3Aa39, each at 5 μg Bt protein per cm^2^ diet. At this concentration, each Bt protein alone is expected to kill approximately 100% of BZ-SS. After 7 days on the treated diet, live larvae that reached the 3rd or later instars were transferred to an untreated diet and reared until pupation. This selection process was repeated for the next three generations (F_3_ to F_5_), and then we selected TRE-RR for the next seven generations using diets treated with Cry1Ac + Cry2Ab + Vip3Aa at 10, 5, and 10 μg Bt protein per cm^2^ diet, respectively. After the seventh generation, we conducted bioassays (see below) to determine responses to each protein individually and in combination.

### Bt proteins

We tested Cry1Ab, Cry1Ac, Cry1A.105, Cry1Fa, Cry2Ab and Vip3Aa protoxins. All were tested as purified protoxins in buffer unless noted otherwise. Bayer CropScience (St. Louis, MO) provided Cry1Ac, Cry1A.105, and Cry2Ab2. For Cry1Ac, we tested lyophilized MVPII powder with 20.0% active ingredient (AI). We used 1.116 mg Cry1A.105 per ml buffer. We used two forms of Cry2Ab. To obtain the LC_50_ values of all strains, we tested lyophilized Bt corn leaf tissue (powder) containing ~ 5.2 mg Cry2Ab per gram. To test for synergy, we used 0.31 mg Cry2Ab per ml buffer. We used the leaf powder to obtain LC_50_ values of Cry2Ab because it was more readily available in the relatively large quantity needed for this purpose. In the synergy tests, we used purified Cry2Ab rather than leaf powder to avoid potential effects of leaf constituents other than Cry2Ab. BASF (Morrisville, NC) provided a liquid formulation containing 3.1 mg Cry1Ab per ml. Corteva Agriscience (Indianapolis, IN) provided Cry1Fa as a lyophilized powder with 53.0% AI. Juan-Luis Jurat-Fuentes, University of Tennessee, provided 2.8 mg Vip3Aa39 (referred to here as Vip3Aa) per ml buffer that was produced by a recombinant *Escherichia coli* strain [51]. The amino acid sequence similarity for Vip3Aa39 is 97.1% with both the Vip3Aa20 protein in Bt corn and Vip3Aa19 in Bt cotton [59].

### Bioassays

To evaluate susceptibility to Bt proteins, we used diet overlay bioassays [20]. We used repeater pipettes to add 0.8 ml liquid *H. zea* diet (Southland Products, Inc. Lake Village, AR) into each well of 128-well bioassay trays (C-D International, Pitman, NJ). After the diet cooled and solidified, we used repeater pipettes to add Bt protoxin suspended in 0.1% Triton-X100 to the diet surface of each well (the total volume of suspension added was 200 μl for Cry2Ab in leaf powder and 40 μl for the other five Bt proteins). After air-drying the Bt suspension, we placed a single neonate (< 24 h old) in each well and covered the wells with vented lids (C-D International, Pitman, NJ). The trays were maintained at 26 ± 1 °C, 50% relative humidity, and a photoperiod of 16:8 h (L:D). Each combination of insect strains by Bt protoxin concentrations was replicated four times, with 16 larvae per replicate. Larval mortality and developmental stage were recorded on the seventh day after infestation. To obtain LC_50_ values, we tested each of the six Bt proteins listed above at 6 to 9 concentrations ranging from 0.01 to 100 μg protoxin per cm^2^ diet plus untreated diet as a control. To evaluate synergy, we tested TRE-RR against Cry1Ac, Cry2Ab2, and Vip3Aa singly and in combinations at either 3.16 or 5.0 μg Bt protoxin per cm^2^ diet. The bioassays comparing single proteins versus combinations to evaluate synergy were started on May 31, 2022 for the lower concentration and June 8, 2022 for the higher concentration.

### Data analysis

We used probit analysis with SAS/STAT v. 9.4 (SAS Institute Inc., Cary, NC) to determine the concentration that killed 50% of larvae (LC_50_) and its 95% fiducial limits (FLs), as well as the slope of the concentration-mortality line and the standard error (SE) of the slope. The number of deaths was adjusted for control mortality [60]. Unless noted otherwise, we considered two LC_50_ values to be significantly different if no overlap occurred between their 95% FLs, which is a conservative criterion [61]. For Cry1Ac and Cry2Ab, which were each tested against BZ-SS on three different dates (October 2020, January 2021, July 2021), we considered an LC_50_ value significantly different between BZ-SS and a Vip3Aa-resistant strain if no overlap occurred between the 95% FLs for the three LC_50_ values for BZ-SS and the 95% FLs for the Vip3Aa-resistant strain, which is a very conservative criterion.

We calculated the resistance ratio (RR) for Vip3Aa as the LC_50_ value for each strain divided by the mean LC_50_ of BZ-SS for Vip3Aa. Unless noted otherwise, we calculated the cross-resistance ratio (CRR) for Cry proteins (against which strains were not selected) as the LC_50_ value for each strain divided by the mean LC_50_ of BZ-SS for the same Cry protein. As an alternative method, we calculated CRRs as the LC_50_ value of each Cry protein for each Vip3Aa-resistant strain divided by the single LC_50_ of each Cry protein for BZ-SS obtained closest to the date when the Vip3Aa-resistant strain was tested against the same Cry protein. For Cry1Ab, Cry1A.105, and Cry1Fa, BZ-SS, these two methods are identical because BZ-SS was tested only once. For Cry1Ac and Cry2Ab, which were each tested against BZ-SS on three different dates, the results from the two methods are similar.

Four of the five Vip3Aa-resistant strains were tested against all five Cry proteins, while one (R2-Vip) was tested against only three, yielding 23 LC_50_ values of Cry proteins for testing the hypothesis that selection with Vip3Aa causes cross-resistance to Cry proteins. Of the 23 LC_50_ values for Cry proteins analyzed here, three were reported previously by Kennedy et al. [48] (for Cry1Ac, Cry1A.105, and Cry2Ab versus LT70-Vip), and 20 are reported here for the first time. Calvin et al. [62] reported the CRRs for TRE-RR for Cry1Ac, Cry2Ab, and Vip3Aa individually, but not the individual LC_50_ values and slopes or results from tests for synergy.

To normalize CRRs, they were log-transformed for statistical analyses. We report means and 95% confidence intervals (CIs) based on the back-transformed results from log-transformed RRs. We used one-sample t-tests of the log-transformed CRRs to test the hypothesis that the CRRs of Cry proteins for Vip3Aa-resistant strains differed significantly from 1, which would occur if selection with Vip3Aa caused positive or negative cross-resistance to Cry proteins.

To evaluate synergy, we used chi-square tests with a continuity correction (http://vassarstats.net/csfit.html) to determine if observed survival differed significantly from expected survival based on the independent action of each Bt protein in a combination. For example, with independent action, expected survival for the combination Cry1Ac + Vip3Aa equals the survival for Cry1Ac alone times the survival for Vip3Aa alone [63]. The control mortality was 0% in the synergy bioassays.

## Supplementary Information

Supplementary Material 1.

## Data Availability

The data generated and analyzed during this study are available from the corresponding author upon reasonable request.

## Abbreviations

AI: Active ingredient
BBMVs: Brush border membrane vesicles
Bt: *Bacillus thuringiensis*
CIs: Confidence intervals
Cry: Crystalline
CRR: Cross-resistance ratio
FLs: Fiducial limits
RH: Relative humidity
RR: Resistance ratio
SE: Standard error
TRE-RR: Triple –resistant
Vip3: Vegetative Insecticidal Protein 3
