# Supplementary material for: Evaluating cross-resistance and synergy between Vip3Aa and Cry proteins from Bt in six strains of Helicoverpa zea derived via F2 screens
Source: Crop Health. 2026 Apr 9;4(1):11. doi: 10.1007/s44297-026-00073-8 (PMC13066062; doi:10.1007/s44297-026-00073-8)
Supplement: Supplementary file 1 — Supplementary Material 1. [file 44297_2026_73_MOESM1_ESM.docx]

**Table S1** Establishment and associated pre-experimental rearing history of the LT70-Vip strain

| **Generation** | **Crossed with BZ-SS** | **Selected with Vip3Aa** | **Description** |
| --- | --- | --- | --- |
| F0-Female |  |  | Female moths collected from light traps Snook, TX, July 2019 |
| F1 |  |  | F1 reared on untreated diet |
| F2(RR): F1*F1 |  | + | F2 selected with Vip3Aa39 3.0 ug per cm sq diet for 7 days to establish RR, and then reared on untreated diet |
| F3(RS): F2(RR)*SS | + |  | Limited F2 survivors crossed with SS to produce RS, and then reared on untreated diet |
| F4(RR): F3(RS)*F3(RS) |  | + | F4 selected with Vip3Aa39 3.0 ug per cm sq diet for 7 days to produce RR, and then reared on untreated diet |
| F5(RR) |  | + | F5 selected with Vip3Aa39 3.16-100 ug per cm sq diet for 7 days, and then reared on untreated diet |
| F6(RS): F5(RR)*SS | + |  | F5 crossed with SS to produce RS, and then reared on untreated diet |
| F7(RR): F6(RS)*F6(RS) |  | + | F7 selected with Vip3Aa39 5.0 ug per cm sq diet for 7 days to produce RR, and then reared on untreated diet |
| F8(RS): F7(RR)*SS | + |  | F7 crossed with SS to produce RS, and then reared on untreated diet |
| F9(RR): F8(RS)*F8(RS) |  | + | F9 selected with Vip3Aa39 5.0 ug per cm sq diet for 7 days to produce RR, and then reared on untreated diet |
| F10(RR) |  |  | F10 reared on untreated diet |
| F11(RS): F10(RR)*SS | + |  | F10 crossed with SS to produce RS, and then reared on untreated diet |
| F12(RR): F11(RS)*F11(RS) |  | + | F12 selected with Vip3Aa39 5.0 ug per cm sq diet for 7 days to produce RR, and then reared on untreated diet |
| F13(RR) |  |  | F13 reared on untreated diet |
| F14(RS): F13(RR)*SS | + |  | F13 crossed with SS to produce RS, and then reared on untreated diet |
| F15(RR): F14(RS)*F14(RS) |  | + | F15 selected with Vip3Aa39 5.0 ug per cm sq diet for 7 days to produce RR, and then reared on untreated diet |
| F16(RR) |  |  | F16 reared on untreated diet |
| F17(RR) |  |  | F17 and after was used for the study |

+ indicates that the resistant strain was crossed with the BZ-SS strain or was selected with Vip3Aa protein.

**Table S2** Establishment and associated pre-experimental rearing history of the M1-Vip strain

| **Generation** | **Crossed with BZ-SS** | **Selected with Vip3Aa** | **Description** |
| --- | --- | --- | --- |
| F0 |  |  | 5th instar larva collected from Cry1Ac + Cry2Ab2 cotton in Alexandria, LA, August 2019 |
| F1: F0-♂ * SS-♀ | + |  | F0 male crossed with SS female to produce F1 progeny, and F1 reared on untreated diet |
| F2(RR): F1*F1 |  | + | F2 selected with Vip3Aa39 3.0 ug per cm sq diet for 7 days to establish RR, and then reared on untreated diet |
| F3(RS): F2 (RR)*SS | + |  | Limited F2 survivors crossed with SS to produce RS, and then reared on untreated diet |
| F4(RR): F3(RS)*F3(RS) |  | + | F4 selected with Vip3Aa39 3.0 ug per cm sq diet for 7 days to produce RR, and then reared on untreated diet |
| F5(RR) |  | + | F5 selected with Vip3Aa39 5.0 ug per cm sq diet for 7 days, and then reared on untreated diet |
| F6(RS): F5(RR)*SS | + |  | F5 crossed with SS to produce RS, and then reared on untreated diet |
| F7(RR): F6(RS)*F6(RS) |  | + | F7 selected with Vip3Aa39 5.0 ug per cm sq diet for 7 days to produce RR, and then reared on untreated diet |
| F8(RS): F7(RR)*SS | + |  | F7 crossed with SS to produce RS, and then reared on untreated diet |
| F9(RR): F8(RS)*F8(RS) |  | + | F9 selected with Vip3Aa39 5.0 ug per cm sq diet for 7 days to produce RR, and then reared on untreated diet |
| F10(RR) |  | + | F10 selected with Vip3Aa39 3.16-100 ug per cm sq diet for 7 days, and survivors were reared on untreated diet |
| F11(RS): F10(RR)*SS | + |  | F10 crossed with SS to produce RS, and then reared on untreated diet |
| F12(RR): F11(RS)*F11(RS) |  | + | F12 selected with Vip3Aa39 5.0 ug per cm sq diet for 7 days to produce RR, and then reared on untreated diet |
| F13(RR) |  |  | F13 reared on untreated diet |
| F14(RS): F13(RR)*SS | + |  | F13 crossed with SS to produce RS, and then reared on untreated diet |
| F15 (RR): F14(RS)*F14(RS) |  | + | F15 selected with Vip3Aa39 5.0 ug per cm sq diet for 7 days to produce RR, and then reared on untreated diet |
| F16 (RR) |  | + | F16 selected with Vip3Aa39 5.0 ug per cm sq diet for 7 days, and then reared on untreated diet |
| F17 (RR) |  |  | F17 reared on untreated diet |
| F18 (RR) |  |  | F18 reared on untreated diet |
| F19(RS): F18(RR)*SS | + |  | F18 crossed with SS to produce RS, and then reared on untreated diet |
| F20 (RR): F19(RS)*F19(RS) |  | + | F20 selected with Vip3Aa39 5.0 ug per cm sq diet for 7 days to produce RR, and then reared on untreated diet |
| F21(RR) |  | + | F21 selected with Vip3Aa39 3.16-100 ug per cm sq diet for 7 days, and survivors were reared on untreated diet |
| F22 (RR) |  |  | F22 and after was used for the study |

+ indicates that the resistant strain was crossed with the BZ-SS strain or was selected with Vip3Aa protein.

**Table S3** Establishment and associated pre-experimental rearing history of the AC4-Vip strain

| **Generation** | **Crossed with BZ-SS** | **Selected with Vip3Aa** | **Description** |
| --- | --- | --- | --- |
| F0 |  |  | 5th instar larva collected from non-Bt corn in Winnsboro, LA, July, 2020 |
| F1: F0-♂ * SS-♀ | + |  | F0 male crossed with SS female to produce F1 progeny, and F1 reared on untreated diet |
| F2(RR): F1*F1 |  | + | F2 selected with Vip3Aa39 3.0 ug per cm sq diet for 7 days to establish RR, and then reared on untreated diet |
| F3(RS): F2 (RR)*SS | + |  | Limited F2 survivors crossed with SS to produce RS, and then reared on untreated diet |
| F4(RR): F3(RS)*F3(RS) |  | + | F4 selected with Vip3Aa39 3.0 ug per cm sq diet for 7 days to produce RR, and then reared on untreated diet |
| F5(RR) |  | + | F5 selected with Vip3Aa39 5.0 ug per cm sq diet for 7 days, and then reared on untreated diet |
| F6(RS): F5(RR)*SS | + |  | F5 crossed with SS to produce RS, and then reared on untreated diet |
| F7(RR): F6(RS)*F6(RS) |  | + | F7 selected with Vip3Aa39 5.0 ug per cm sq diet for 7 days to produce RR, and then reared on untreated diet |
| F8(RR) |  |  | F8 reared on untreated diet |
| F9(RS): F8(RR)*SS | + |  | F8 crossed with SS to produce RS, and then reared on untreated diet |
| F10(RR): F9(RS)*F9(RS) |  | + | F10 selected with Vip3Aa39 5.0 ug per cm sq diet for 7 days to produce RR, and then reared on untreated diet |
| F11(RR) |  |  | F11 reared on untreated diet |
| F12(RS): F11(RR)*SS | + |  | F11 crossed with SS to produce RS, and then reared on untreated diet |
| F13(RR): F12(RS)*F12(RS) |  | + | F13 selected with Vip3Aa39 5.0 ug per cm sq diet for 7 days to produce RR, and then reared on untreated diet |
| F14(RR) |  |  | F14 reared on untreated diet |
| F15(RR) |  | + | F15 selected with Vip3Aa39 3.16-100 ug per cm sq diet for 7 days, and survivors were reared on untreated diet |
| F16(RR) |  |  | F16 and after was used for the study |

+ indicates that the resistant strain was crossed with the BZ-SS strain or was selected with Vip3Aa protein.

**Table S4** Establishment and associated pre-experimental rearing history of the R2-Vip strain

| **Generation** | **Crossed with BZ-SS** | **Selected with Vip3Aa** | **Description** |
| --- | --- | --- | --- |
| F0 |  |  | 5th instar larva collected from Cry1Ab sweet corn in Stoneville, MS, July, 2020 |
| F1: F0-♂ * SS-♀ | + |  | F0 male crossed with SS female to produce F1 progeny, and F1 reared on untreated diet |
| F2(RR): F1*F1 |  | + | F2 selected with Vip3Aa39 3.0 ug per cm sq diet for 7 days to establish RR, and then reared on untreated diet |
| F3(RS): F2 (RR)*SS | + |  | Limited F2 survivors crossed with SS to produce RS, and then reared on untreated diet |
| F4(RR): F3(RS)*F3(RS) |  | + | F4 selected with Vip3Aa39 3.0 ug per cm sq diet for 7 days to produce RR, and then reared on untreated diet |
| F5(RR) |  | + | F5 selected with Vip3Aa39 5.0 ug per cm sq diet for 7 days, and then reared on untreated diet |
| F6(RS): F5(RR)*SS | + |  | F5 crossed with SS to produce RS, and then reared on untreated diet |
| F7(RR): F6(RS)*F6(RS) |  | + | F7 selected with Vip3Aa39 5.0 ug per cm sq diet for 7 days to produce RR, and then reared on untreated diet |
| F8(RR) |  |  | F8 reared on untreated diet |
| F9(RS): F8(RR)*SS | + |  | F8 crossed with SS to produce RS, and then reared on untreated diet |
| F10(RR): F9(RS)*F9(RS) |  | + | F10 selected with Vip3Aa39 5.0 ug per cm sq diet for 7 days to produce RR, and then reared on untreated diet |
| F11(RR) |  |  | F11 reared on untreated diet |
| F12(RS): F11(RR)*SS | + |  | F11 crossed with SS to produce RS, and then reared on untreated diet |
| F13(RR): F12(RS)*F12(RS) |  | + | F13 selected with Vip3Aa39 5.0 ug per cm sq diet for 7 days to produce RR, and then reared on untreated diet |
| F14(RR) |  | + | F14 selected with Vip3Aa39 3.16-100 ug per cm sq diet for 7 days, and survivors were reared on untreated diet |
| F15(RR) |  |  | F15 and after was used for the study |

+ indicates that the resistant strain was crossed with the BZ-SS strain or was selected with Vip3Aa protein.

**Table S5** Establishment and associated pre-experimental rearing history of the R15-Vip strain

| **Generation** | **Crossed with BZ-SS** | **Selected with Vip3Aa** | **Description** |
| --- | --- | --- | --- |
| F0 |  |  | 5th instar larva collected from Cry1Ab sweet corn in Stoneville, MS, July, 2020 |
| F1: F0-♂ * SS-♀ | + |  | F0 male crossed with SS female to produce F1 progeny, and F1 reared on untreated diet |
| F2(RR): F1*F1 |  | + | F2 selected with Vip3Aa39 3.0 ug per cm sq diet for 7 days to produce RR, and then reared on untreated diet |
| F3(RS): F2 (RR)*SS | + |  | Limited F2 survivors crossed with SS to produce RS, and then reared on untreated diet |
| F4(RR): F3(RS)*F3(RS) |  | + | F4 selected with Vip3Aa39 3.0 ug per cm sq diet for 7 days to produce RR, and then reared on untreated diet |
| F5(RR) |  | + | F5 selected with Vip3Aa39 5.0 ug per cm sq diet for 7 days, and then reared on untreated diet |
| F6(RS): F5(RR)*SS | + |  | F5 crossed with SS to produce RS, and then reared on untreated diet |
| F7(RR): F6(RS)*F6(RS) |  | + | F7 selected with Vip3Aa39 5.0 ug per cm sq diet for 7 days to produce RR, and then reared on untreated diet |
| F8 (RR) |  |  | F8 reared on untreated diet |
| F9(RS): F8(RR)*SS | + |  | F8 crossed with SS to produce RS, and then reared on untreated diet |
| F10(RR): F9(RS)*F9(RS) |  | + | F10 selected with Vip3Aa39 5.0 ug per cm sq diet for 7 days to produce RR, and then reared on untreated diet |
| F11(RR) |  |  | F11 reared on untreated diet |
| F12(RS): F11(RR)*SS | + |  | F11 crossed with SS to produce RS, and then reared on untreated diet |
| F13(RR): F12(RS)*F12(RS) |  | + | F13 selected with Vip3Aa39 5.0 ug per cm sq diet for 7 days to produce RR, and then reared on untreated diet |
| F14(RR) |  | + | F14 selected with Vip3Aa39 3.16-100 ug per cm sq diet for 7 days, and survivors were reared on untreated diet |
| F15(RR) |  |  | F15 and after was used for the study |

+ indicates that the resistant strain was crossed with the BZ-SS strain or was selected with Vip3Aa protein.
